# Use of enantiomeric properties of sodium chlorate to assess primary and secondary nucleation under sonication

**DOI:** 10.1016/j.ultsonch.2021.105763

**Published:** 2021-09-22

**Authors:** Conor W. Copithorne-Crainey, Fraser J. Armstrong, Madeleine Bussemaker, Judy Lee

**Affiliations:** Department of Chemical and Process Engineering, University of Surrey, Guildford, Surrey, GU2 7XH, United Kingdom

**Keywords:** Sonocrystallization, Primary nucleation, Cavitation bubbles, Chiral Crystallization, Sonoluminescence

## Abstract

Ultrasound is known to promote crystal nucleation, but despite significant research there remains uncertainty about how the mechanisms are affected. Despite the proposal of various primary nucleation theories, most studies provide no way to quantify or observe the extent to which primary nucleation is taking place, leaving open the possibility that sonocrystallisation is occurring by a secondary nucleation-driven mechanism. By utilising the widely reported enantiomeric properties of sodium chlorate, the extent to which ultrasound can induce primary nucleation can clearly be observed. It was demonstrated during seeded cooling crystallisation that when stirring the seed similarity was 99.3% on average, indicating secondary nucleation had almost exclusively taken place. The application of ultrasound however, decreased the seed similarity to 85.8% and 92.4% when applying 98 kHz and 200 kHz ultrasound respectively, clearly showing that primary nucleation had been induced and indicating the frequency dependency of the induced primary nucleation. This frequency dependency suggests a link between crystal nucleation and high intensity cavitation collisions and collapses, and the potential existence of a collapse/collision intensity threshold required to induce primary nucleation. In addition, secondary nucleation rate was investigated using anti-solvent crystallisation and was observed to increase with the application of ultrasound, though it appeared frequency independent (between 98 kHz & 200 kHz), suggesting that higher energy cavitational events are less important in inducing secondary nucleation or that a lower cavitation intensity threshold exists compared to primary nucleation.

## Highlights

• Evidence short sonication time induces primary nucleation.
• More primary nucleation events at 98 kHz compared to 200 kHz.
• Primary nucleation requires more intense cavitational collapse bubbles.
• 98 kHz and 200 kHz induced the same secondary nucleation rate.

## 1 Introduction

Crystallisation is a commonly used purification and separation technique utilised across the pharmaceutical, food and fine chemical industries [1], [2], [3], whereby solid solute particles precipitate out of a solution as the system tries to reach an equilibrium saturated state. The driving force for crystallisation is supersaturation of solute, which can be generated by cooling or evaporating solvent from a saturated solution or by the addition of an antisolvent. Mechanistically this involves two steps, namely crystal nucleation (the “birth” of new crystals) and crystal growth [4], [5]. The process is stochastic (or random) which can make it challenging to control and to predict the final crystal properties [6]. The application of ultrasound (US), mechanical sound waves above the audible frequency of 20 kHz [7], has been shown to greatly influence and improve the crystallisation process [8], [9] which has led to significant interest and study of sonocrystallisation or ultrasound enhanced crystallisation. The effects caused by sonocrystallisation are a result of the acoustic cavitation phenomenon induced by ultrasound [10], in which oscillating pressures created by the sound waves cause the growth and collapse of microbubbles [11]. This acoustic cavitation also causes the phenomenon of sonoluminescence [11], [12], [13], [14], [15], [16], the process by which the intense collapse of the cavitation bubbles causes a short burst of light to be emitted alongside high temperatures and pressures [17]. This has become a useful analytical tool to quantify the amount of acoustic cavitation taking place [10].

Numerous groups report the positive impact of ultrasound, including significantly reducing the induction time [18], [19], [20], [21], [22], [23], reducing the metastable zone width (MSZW) [20], [21], [22], [23], [24], [25], [26], [27] and improving the rate of formation of new nuclei [27], [28]. In addition, ultrasound has been shown to control final crystal properties such as the crystal size distribution [29], [30], [31], [32], [33], [34], [35], crystal polymorphism [34], [36], [37], [38] and crystal chirality [39], [40], [41], [42], [43] – all of which are very important to control, particularly in the pharmaceutical and food industries. Given the complexity of ultrasonic cavitation and traditional crystallisation and the various factors that impact both processes, and despite a significant body of research investigating ultrasound’s effect on the crystallisation process, the detailed mechanisms involved remain elusive. In particular the nucleation mechanisms that occur and are favoured by ultrasound are uncertain.

There are two types of nucleation, primary and secondary, and these can be broken down into further subgroups, with ultrasound affecting each in different ways. Primary nucleation occurs in the absence of existing crystalline material in the system and is further split into homogeneous and heterogeneous nucleation. Homogeneous nucleation occurs in the absence of any foreign particles/surfaces in the solution and happens spontaneously, whilst heterogeneous nucleation is enabled by foreign surfaces in the solution [4]. Notably it has been proposed that cavitation bubbles can act as nucleation centres thereby inducing heterogeneous nucleation, given the bubbling of air through a supersaturated solution resulted in a similar reduction in MSZW to that observed during sonocrystallisation [25]. In addition, studies by Guo et al. [44] have suggested that ultrasound reduces the energy barrier for heterogeneous nucleation to occur by reducing the correction factor, a term in the fundamental classical nucleation equations [5]. It is proposed this could occur as a result of nucleation taking place at a bubble/solution interface, a bubble/solution/foreign solid surface interface, or as a result of a shockwave, following the collapse of a cavitation bubble, changing the shape of a crystal cluster [44]. In the homogenous regime, it has been suggested that ultrasound enhances the nucleation rate through increasing turbulences in the system through shockwaves and acoustic streaming [8], [22]. Whilst numerous other homogeneous nucleation theories have been proposed, such as the cooling [45], pressure [45], and segregation theory [46], recent studies have placed doubts upon these hypotheses, as discussed by Jordens et al. [8]. More recent studies by Lee et al. have suggested the link between non-symmetrical cavitational activity and crystal nucleation [10] and a potential modification to the classical nucleation theory to account for the additional energy supplied to induce primary nucleation from high energy bubble collapses and collisions [18].

The mechanisms behind ultrasound enhanced secondary nucleation are less studied. Secondary nucleation occurs when new crystals form from pre-existing crystalline material in the solution and, compared to primary nucleation, can occur at much lower levels of supersaturation [4]. It is believed that ultrasound induced secondary nucleation mainly occurs as a result of fragmentation and attrition of existing crystals with the fragments proceeding to act as new nucleation sites [47]. This has been visually observed in the cooling crystallisation of ice with high-speed images [47], [48], [49], whilst modelling has also demonstrated the importance of crystal breakage during sonocrystallisation [27].

Chiral conglomerate crystals, such as sodium chlorate, have been used throughout literature as model compounds to investigate the type of nucleation that occurs in a given system. First reported and used by Denk & Botsaris in 1972 [50], [51], the enantiomeric properties of sodium chlorate can be used as a “tag” to determine whether crystals formed during a crystallisation process originated from the solution by primary nucleation or from a seed crystal by secondary nucleation. This process was first adapted for use with ultrasound by Song et al. in 2008, who reported observing chiral symmetry breaking with unseeded cooling crystallisation of sodium chlorate resulting in a large enantiomeric imbalance (whilst seeded crystallisation resulted in enantiomeric pure crystals matching the seed) [40]. Later work concluded that this symmetry breaking was a result of a self-seeding effect with catastrophic secondary nucleation causing chiral amplification of the initial enantiomeric imbalance caused once the first crystal formed spontaneously [39]. Whilst this work indicated the dominance of secondary nucleation, given the long sonication times, high ultrasonic powers and seeding style used, other processes may have played a part such as the recently reported phenomenon of Viedma ripening (attrition-enhanced deracemisation) [52]. During this process continuous grinding can transform a mixture of mixed chirality crystals to a single chirality through a continuous dissolution-crystallisation process, thereby autocatalytically amplifying an initial enantiomeric imbalance. [41], [52]. As a result, while Song et al.’s results [40] demonstrate ultrasound’s ability to significantly enhance the secondary nucleation rate, they do not indicate that ultrasound is unable to induce primary nucleation.

While significant discussion has taken place surrounding ultrasound’s effect on primary nucleation, with numerous mechanisms proposed [53], to the authors’ knowledge there remains a lack of conclusive evidence that primary nucleation is actually taking place. It has even been previously reported that sonocrystallisation occurs through a secondary nucleation pathway [54], [55]. The present work involves new seeded experiments carried out with sodium chlorate to determine the impact of ultrasound on both primary and secondary nucleation. In contrast to the previously reported studies, the key focus of the seeded study was placed on nucleation induced early on, with a fixed supersaturation and a comparison made to a non-sonicated system. Furthermore, a more controlled system was used with the suspension of a seed crystal for a fixed amount of time before being removed, similar to the procedure used by Callahan et al. to compare two non-sonicated stirred crystallisers [56]. As a result, this study aims to demonstrate conclusively, for the first time, ultrasound’s ability to induce primary nucleation, alongside secondary nucleation under two different frequencies, 98 kHz and 200 kHz. Finally, through an anti-solvent crystallisation study, the impact of ultrasonic frequency on the secondary crystallisation rate will also be discussed.

## 2 Experimental details

### 2.1 Materials

Sodium chlorate (NaClO_3_, 99%+, ACROS Organics^TM^), absolute ethanol (EtOH, 99.99%) and sodium chloride (NaCl, 99.75%) were purchased from Fisher Scientific. Purified water from a Milli-Q system was used for all experiments (resistivity of 18.2 MΩ cm at 25 °C). Whatman glass fibre filter paper (Grade GF/C, nominal pore size 1.2 μm) and Whatman polyamide membrane filters (0.45 μm) were used, as discussed in the respective sections.

### 2.2 Equipment

**Fig. 1:**
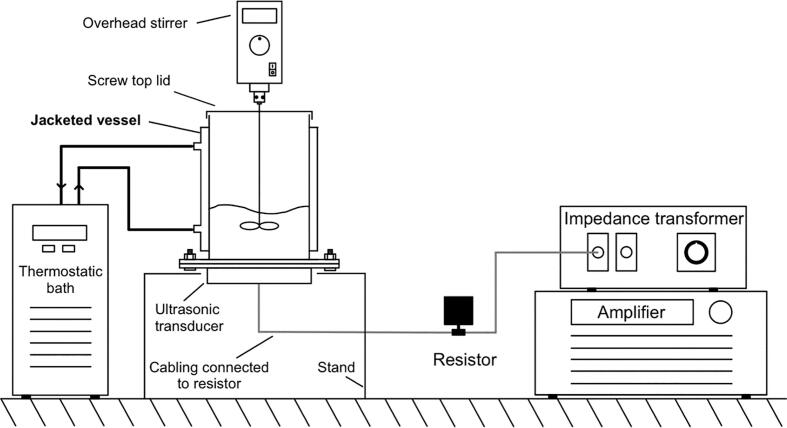
Schematic diagram of the experimental set up used across the various studies. On the left is the jacketed experimental reactor, connected thermostatic bath, and overhead stirrer. On the right, the amplifier is connected to the impedance transformer which is connected in series to a 10kΩ resistor and the ultrasonic transducer.

A schematic of the experimental setup used in the crystal nucleation study, crystallisation rate study and sonoluminescence imaging is shown in Fig. 1.

The nucleation study used a jacketed glass reactor (inner diameter and height measured 64 mm and 180 mm, respectively) as pictured in Fig. 1, with a screw top lid (with a hole for an overhead stirrer) and an ultrasonic transducer fitted at the bottom. To control the temperature, water was circulated through the reactor cooling jacket, by a Julabo thermostatic bath. The anti-solvent crystallisation study used the same experimental set-up, however given the short sonication times, the water jacket and lid were not used. For the sonoluminescence imaging the water jacket was emptied and the stirrer removed.

Two different transducers, made by Honda Electronics Co. LTD., were used: a multi-frequency plate Langevin transducer operated at 98 kHz and a 200 kHz plate transducer (which consisted of a 5 cm diameter piezo-electric ceramic adhered to a 10 cm stainless steel vibration plate). The signal to the transducer was delivered by a T&C Power Conversion AG 1006 amplifier, connected in series to a T series low frequency RF impedance transformer (T05 LF-7 model from T&C Power Conversion) and a 10 kΩ resistor. This model of amplifier allows monitoring of the forward, load and reflected power and was operated in load power mode which automatically increased power to compensate for any reflected power. Reflected power was minimised by tuning the frequency and the use of the impedance transformer. An additional impedance matcher was used with the multi-frequency transducer, when set to 98 kHz, to reduce excess reflected power.

The calorimetric power of the two transducers driven at 20 W amplifier power was investigated by measuring the temperature increase of 100 g of water over 6 min, with measurements taken every 30 s. Using a specific heat capacity of 4180 J/(kg.°C) the calorimetric power was then calculated. The measured calorimetric power was 5.3 W and 8.1 W for the 98 kHz and 200 kHz, respectively. For amplifier power of 1 W and 98 kHz, the calorimetric power was 0.27 W.

### 2.3 Sonoluminescence imaging

Sonoluminescence analysis was carried out to determine the intensity and location of cavitational activity in the reactor. The equipment described in Fig. 1 was moved to a dark box and the spatial distribution and light quantification of emitted sonoluminescence was then captured with an ANDOR iXon3 EMCCD low light camera and software. The camera operated at − 70 °C and applied an EM gain level of 50 and exposure time of 5 s. A background intensity was first captured, followed by an image after 10 s of pre-sonication. Images were taken of the reactor with 400 ml water and again with 738 g/L of aqueous sodium chlorate solution, when applying 20 W (amplifier power) of 98 kHz and 200 kHz ultrasound. The raw data was exported to Microsoft Excel, and the total integrated intensity for each condition was calculated as the difference between the sonicated and silent images.

### 2.4 Seed similarity study

#### 2.4.1 Crystal chirality analysis

**Fig. 2:**
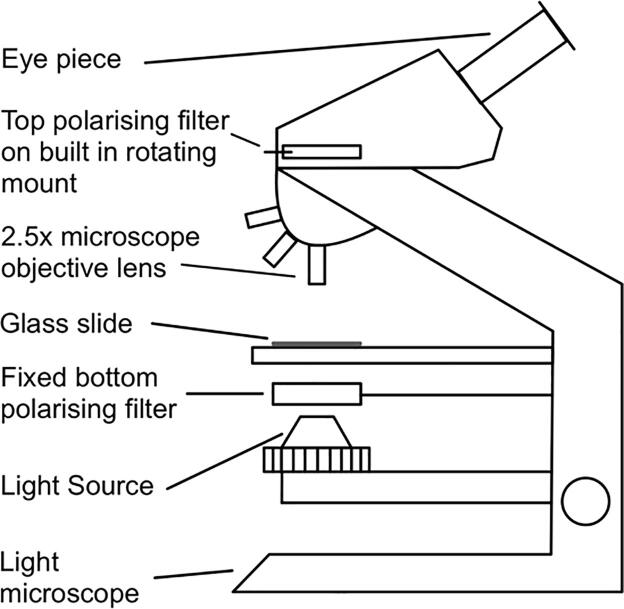
Schematic diagram of the polarimeter used to test all crystals.

Sodium chlorate is an inorganic salt which is racemic in solution, but upon crystallisation forms chiral conglomerate crystals. Two enantiomers can form, dextrorotatory and levorotatory crystals [56], referred to throughout this paper as left- and right-handed crystals, respectively. These crystals display optical activity and, as such, the handedness of the crystals can be determined by their direction of rotation of plane polarised light, a property that has been utilised throughout a number of previous studies [40], [50], [56], [57]**.** A polarimeter, in this case a bottom-lit microscope with polarising filters above and below the crystals, as represented in Fig. 2, was used to determine each crystal’s handedness by rotating the top filter clockwise or anticlockwise via the rotating mount.

**Fig. 3:**
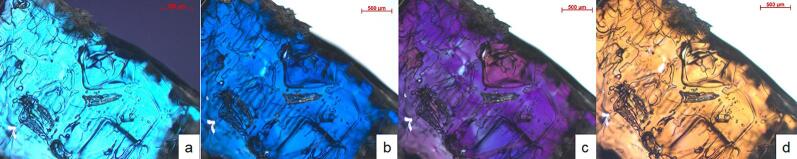
Colour sequence observed in a polarimeter for both left- and right-handed sodium chlorate crystals depending on the direction of rotation of the top polarising filter as discussed in the text.

When the polarisers are initially crossed (perpendicular to each other), the background is at its darkest whilst both the left- and right-handed crystals are a pale blue colour (Fig. 3.a). When rotating the top polarising filter clockwise the right-handed (dextrorotatory) crystals appeared dark blue (Fig. 3.b); and with further rotation the right-handed crystals became dark purple (Fig. 3.c) followed by light orange (Fig. 3.d). During this clockwise rotation the left-handed (levorotatory) crystals remained a bright pale blue. On the other hand, when rotating the top polarising filter anti-clockwise the colour sequence discussed above was observed for all left-handed crystals whilst the right-handed crystals remained pale blue.

#### 2.4.2 Seed crystal growth

To obtain the seed crystals a 738 g/L sodium chlorate in Milli-Q water solution was prepared and held at 40 °C in a shaking water bath, to ensure dissolution, before being transferred to an open topped beaker and left to crystalise at room temperature for ∼20 h. The resulting crystals were then extracted by filtration (Buchner funnel & Whatman glass fibre filter paper) and immediately rinsed with Milli-Q water to remove traces of mother liquor before being dried between pieces of fresh filter paper. All seed crystals were then analysed (as discussed in Section 2.2) to determine their handedness before being used throughout the seeded crystal nucleation study as discussed in Section 2.4.2 below. Similar sized crystals and a mixture of both handed crystals were selected.

#### 2.4.3 Seeded crystal nucleation

**Fig. 4:**
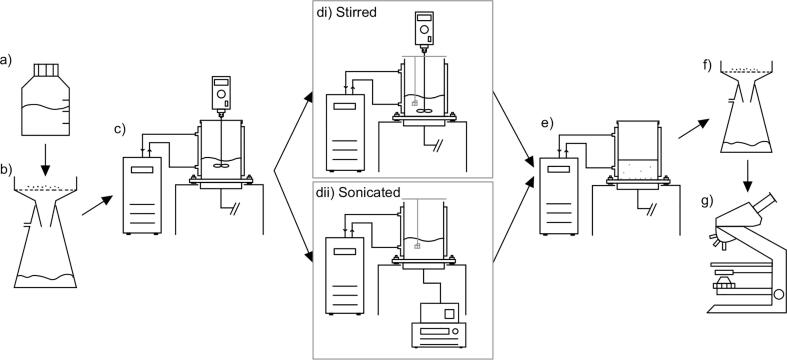
Summary of the methodology used in the nucleation study: a) Sodium chlorate dissolution. b) Impurity filtration. c) Transfer to reactor & cooling. di) Route taken when stirring and suspending the seed crystal (with no US) - crystal suspended for 5 min under 300 rpm agitation. dii) Route taken when sonicating and suspending the seed crystal (with no stirring) – crystal suspended for 5 min under 20 W 98/200 kHz ultrasound. e) Crystal growth following seed removal. f) Crystal recovery via filtration. g) Crystal analysis as discussed in Section 2.4.1.

Fig. 4 shows the experimental procedure followed in this study to induce and then analyse sodium chlorate crystals. 400 ml of sodium chlorate-water solution (738 g/L or 0.993g_NaClO3_/g_water_) was made-up in a Duran bottle and held at 40 °C in a shaking water bath for 1 h to guarantee complete dissolution. The solution was then filtered using a Büchner funnel & flask with Whatman glass fibre filter paper to remove any foreign particles entrained in the solution. The solution was immediately transferred to the jacketed reactor and held at 40 °C under agitation for a further 30 min to ensure no crystallisation occurred as a result of cooling during the filtration step. The solution was steadily cooled over 30 min to 29 °C by lowering the water jacket temperature.

A single seed crystal of known handedness was attached to a wire and thoroughly washed with Milli-Q water to remove any crystalline fines (care was taken to use similar sized seeds, by eye, for each experiment, although a degree of variability would have been introduced in this step). The wire was then attached to a bar which was placed across the vessel opening to suspend the crystal within the solution. The seed was suspended slightly off to one side to avoid interference with the stirrer in the stirred experiments and placed in the same location in the sonicated experiments for continuity. As soon as the seed crystal was suspended the stirrer or ultrasound was started (300 rpm stirrer speed or 20 W amplifier power of 98/200 kHz ultrasound) and left for 5 min. Immediately afterwards the ultrasound/stirring was stopped, the seed crystal was removed, and the crystalliser was sealed and left at 29 °C undisturbed overnight. This allowed any crystals that had formed to grow to a measurable size.

After 20–22 h the crystals were removed from the crystalliser by filtration before being dried with fresh filter paper. The left- and right-handed crystals were separated using the analysis method discussed in Section 2.4.1 and weighed to determine their respective percentages, allowing the seed similarity to be calculated. Each experiment was repeated at least 4 times in order to establish an average, and to account for the large variability observed.

### 2.5 Anti-Solvent crystallisation rate study

Sodium chlorate stock solution (0.5g_NaClO3_/g_Water_) was made up in a Duran bottle by mixing salt and Milli-Q water and left in a shaker at room temperature for 30 min. Starting with 100 g absolute ethanol at room temperature in the glass reactor (stirred at 300 rpm with an overhead stirrer), 10 g sodium chlorate stock solution was poured into the vessel. At the same time the ultrasound (when appropriate) and the stopwatch were started. 5 ml samples were taken at different time points using a pipette and the aliquot was immediately filtered using a Büchner funnel (with a Whatman polyamide membrane filter) in order to retain only the crystal mass in the sample at the given time. The filter paper was then dried, and the mass was taken to determine how the crystal mass changed with time. This experiment was carried out with low power ultrasound (98 kHz, 1 W), high power ultrasound (98 kHz & 200 kHz, 20 W) and under stirred only conditions without sonication. Each time point was repeated at least 3 times to obtain an average.

## 3 Results and discussions

A number of studies were carried out to investigate ultrasound’s effects on the crystallisation process. Firstly, sonoluminescence imaging was carried out to determine the cavitation activity in sodium chlorate solution at different sonication frequencies. A seed similarity study was then undertaken to determine the initial nucleation mechanisms taking place during the sonicated cooling crystallisation of sodium chlorate under two different ultrasonic frequencies. In addition, using an anti-solvent sodium chlorate and ethanol system, the effect of ultrasound on the secondary crystal nucleation rate was studied.

### 3.1 Sonoluminescence imaging

**Fig. 5:**
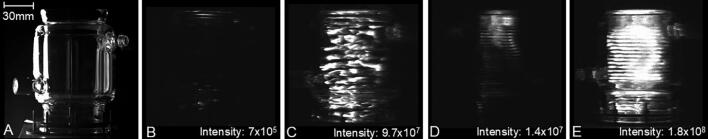
(A) shows the experimental reactor when lit. Sonoluminescence images and intensities when applying: (B) 98 kHz US to water; (C) 98 kHz US to NaClO_3_ solution; (D) 200 kHz US to water and (E) 200 kHz US to NaClO_3_ solution. 20 W amplifier power has been applied to all sonicated systems, but the measured calorimetric power was 5.3 W and 8.1 W for the 98 kHz and 200 kHz, respectively.

Sonoluminescence imaging was used as a way to quantify and demonstrate that cavitation. Fig. 5A shows the experimental reactor, and Fig. 5B to E show the multibubble sonoluminescence (MBSL) emitted when applying 98 kHz and 200 kHz ultrasound to a water and sodium chlorate solution. It is clearly demonstrated that a much higher MBSL is observed with the sodium chlorate solution compared to water for both frequencies. This agrees with previously reported literature, where an increasing salt concentration exhibits increased MBSL, as a result of a decreased gas solubility at higher salt concentrations, while above a critical salt concentration the MBSL decreases [58], [59].

The results also show that, while substantial sonoluminescence is emitted at 98 kHz with sodium chlorate in Fig. 5C, the sonoluminescence captured in Fig. 5E at 200 kHz is considerably higher. Whilst lower frequencies result in individual bubbles exhibiting higher collapse intensities given their larger size [60], the number of bubbles present in a system increases with higher frequencies as the number of bubbles is highly influenced by the number of ultrasonic antinodes present [37], [61]. Higher frequencies directly result in lower wavelengths, and thus more antinodes that are much closer together. This can clearly be observed in Fig. 5E by the large number of bands in the reactor. As a result, while the sonoluminescence emitted from a single bubble at 98 kHz would be expected to be higher, the overall sonoluminescence emitted in the multi bubble system is significantly higher at 200 kHz. This is in line with previously reported literature where a maximum sonoluminescence intensity is repeatably found in the mid kHz range, as a result of this balance between collapse intensity and number of bubbles [10], [61], [62]. The higher intensity observed at 200 kHz could also be attributed to a slightly higher calorimetric power measured.

### 3.2 Seed similarity study to probe primary and secondary nucleation

**Fig. 6:**
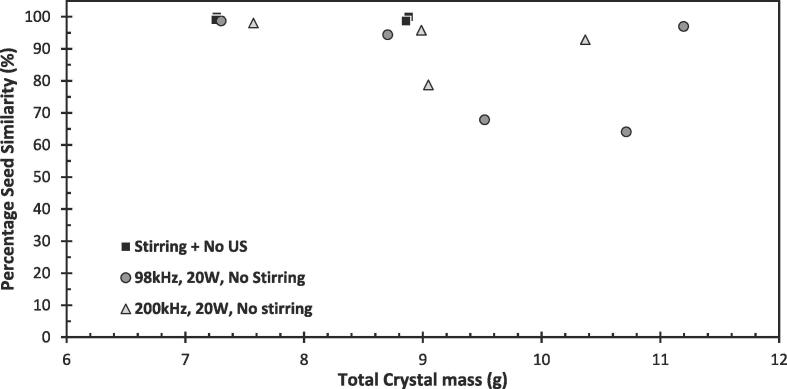
Results for the nucleation study demonstrating the percentage seed similarity against the total mass of product crystals for the three conditions investigated: Stirred only case; 5.3 W at 98 kHz ultrasound only and 8.1 W at 200 kHz ultrasound only. Each point represents a separate experiment.

Fig. 6 shows the variation in the mass of the crystals formed and percentage seed similarity obtained during the seed similarity study for the three conditions investigated: stirring; 98 kHz US and 200 kHz US. The variation in the total crystal mass yield across each experiment is likely to be a result of slight temperature variation overnight between experiments and not a result of any sonication/stirring. The seeding stage only took place for 5 min, at which point only microscopic crystals had formed. Given nucleation was not readily observed under silent, unseeded conditions and low supersaturation ratio of 1.03 used, following the removal of the seed crystal no further nucleation is expected. Therefore, the total crystal mass is a result of the slow crystal growth that occurs over the following 20–22 h.

It is clear from Fig. 6 that stirring during seed suspension consistently resulted in product crystals with an almost 100% similarity to the seed handedness and very low variation in seed similarity between studies, as observed by the low standard deviation of 0.66% (Table 1). Given that these crystals almost exclusively bore the same handedness as the seed, it would suggest the nucleation mechanism was almost solely secondary with all product crystals being derived from the singular parent seed crystal (the handedness of which is given in the supplementary information for each experiment in Table S1 in Supplementary Information).

**Table 1:** Summary of average crystal mass and percentage seed similarity average and standard deviation for the three conditions investigated in the nucleation study.

Condition	Average Crystal Mass (g)	Percentage Seed Similarity (%)	
		Average	Standard Deviation
Stirring + No US	8.332	99.26	0.66
98 kHz, 5.3 W, No Stirring	8.906	85.75	16.94
200 kHz, 8.1 W, No stirring	8.994	91.40	8.68

On the other hand, the application of ultrasound resulted in significant variation in percentage seed similarity, with an average of 91.4 ± 8.7% and 85.8 ± 16.9% for the respective 200 kHz and 98 kHz experiments. The large standard deviation in seed similarities observed in the sonicated studies highlights the stochastic nature of primary nucleation. Although secondary nucleation remains the dominant mechanism with the application of ultrasound, given the reduction in seed similarity compared to the stirred only system, some primary nucleation must have occurred to produce crystals with the opposite handedness to the seed. When a primary nuclei forms, there is a 50:50 chance it will share the same handedness as the seed crystal. Secondary nucleation will also be enhanced simultaneously, amplifying the chiral imbalance (as was observed by Song et al. to a more significant extent [39]) caused by the presence of the seed crystal and any primary nuclei which formed early on as a result of sonication. The initial balance between primary and secondary nucleation rates, and the random nature of any primary nuclei sharing or not sharing the seed crystal’s chirality, would inevitably result in variation in seed similarity across multiple studies. This outcome demonstrates that ultrasound itself was able to induce primary nucleation, where stirring alone was not.

#### 3.2.1 Differences & explanations to previously reported studies

In 2008 Song et al. had also used a sodium chlorate-based procedure to investigate ultrasounds effects on chirality and reported observing chiral symmetry breaking with the unseeded cooling sonocrystallisation of sodium chlorate, resulting in crystals mainly of a single, but randomly selected, chirality (>96%) [40]. They attributed the result to cavitation-induced secondary nucleation, amplifying an initial enantiomeric imbalance caused by the initial stochastic formation of a nucleus. Furthermore, in seeded studies the enantiomeric purity was observed to increase to 100% [40]. The work suggested the dominance of secondary nucleation and supported a secondary nucleation-dominated mechanism for sonocrystallisation processes in general.

While the observation of primary nucleation reported in this work appears to differ from Song et al.’s seeded study [40], there are some notable differences between the two experiments. In particular, Song et al. used a much higher ultrasonic power of 50 W with a 40 kHz ultrasonic bath [40], compared to the 20 W amplifier power of 98/200 kHz ultrasound used in this study. Song et al. naturally cooled the sodium chlorate solution (saturation temperature of 50 °C) from 75 °C until the solution became supersaturated, all while applying continuous high-powered ultrasound. In addition, the seed was introduced into the unsaturated solution at 75 °C, just after the ultrasonic field was applied. Given the free-energy barrier for secondary nucleation is lower than that for primary nucleation, secondary nucleation can occur at lower levels of supersaturation (and therefore a lower degree of subcooling) [4]. As a result, with a slow and continuous reduction in temperature, whilst applying continuous ultrasound in the presence of a seed crystal, one would expect secondary nucleation to take place first and to dominate, before a large enough level of supersaturation (lower temperature) was reached that could have allowed primary nucleation to occur.

Song et al. also observed many crystals crashing out of solution at once following 10–16 °C of subcooling, dubbed “catastrophic secondary nucleation”. At this point ultrasound was applied for a further 20 min. As a result, the phenomenon of Viedma ripening, or attrition-enhanced deracemisation (where a conglomerate crystal mixture of mixed chirality is able to entirely transform to a single chirality) may have influenced the final chiral distribution [52], [63]. Small fragments created during continuous abrasion-grinding (traditionally achieved by bead grinding) can dissolve and then contribute to the growth of larger crystals through Ostwald ripening or agglomeration, thereby “feeding” the larger crystals [52]. This dissolution-crystallisation phenomenon continues to amplify the enantiomer in excess autocatalytically until complete chiral purity is achieved [52]. Given ultrasound’s ability to cause sonofragmentation (ultrasound-induced breakage), it has been suggested as an alternative to bead grinding for inducing Viedma ripening [64]. A recent study by Xiouras et al. using sodium chlorate, demonstrated ultrasound’s ability to quickly form submicron particles by abrasion leading to fast deracemisation [41], [43]. While complete deracemisation was not achieved with ultrasound alone, this was a result of an insufficient size reduction of the larger “counter-enantiomer” crystals given sonofragmentation took place by abrasion rather than by fracture [43]. As a result, a number of larger “counter-enantiomer” crystals remained, preventing enantiomeric purity from being reached [43]. In Song et al.’s study however, ultrasound was applied immediately and throughout the crystallisation process and therefore there would have been no opportunity for crystals to grow to a significant size [40]. One would expect no limitation to reaching enantiomeric purity, with the continuous application of high-power and low frequency ultrasound throughout able to maintain a small crystal size distribution, as has been observed in numerous other studies [30], [31]. It is therefore very plausible that the phenomenon of Viedma ripening may have provided the final push towards enantiomeric purity observed in Song et al.’s study.

In contrast, in the study reported herein, a supersaturated state was first reached before seeding and sonication took place. A fixed temperature of 29 °C was maintained throughout the study and the suspension of the seed crystal took place for a fixed 5 min under stirring/ultrasound before being removed. As a result, the final crystals that formed gave an indication of the initial nucleation that took place and any effects of Viedma ripening, and ultrasound-enhanced chiral amplification would have been significantly less prevalent.

### 3.3 Anti-Solvent crystallisation rate to probe secondary nucleation

**Fig. 7:**
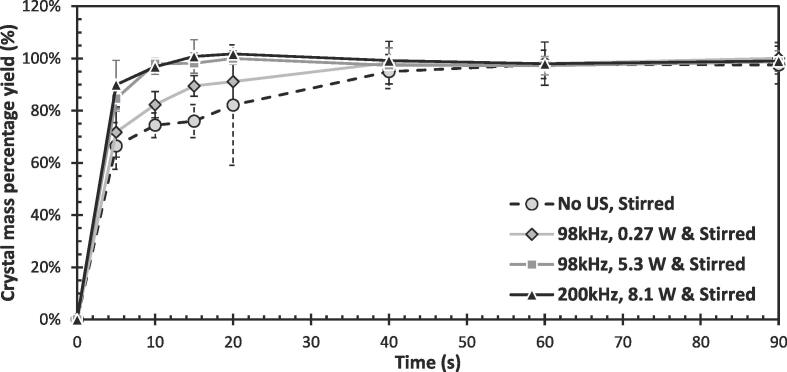
Anti-solvent crystal mass percentage yield as a function of time, for sonicated & stirred and stirred-only experiments. All studies were stirred at 300 rpm and the power quoted is the calorimetric power.

Fig. 7 shows the effect of stirring and the application of ultrasound on the change in percentage crystal yield with time in the anti-solvent sodium chlorate system. The change in percentage crystal yield is a direct indicator of the crystallisation rate and crystal growth. However, the latter can be assumed to be negligible as Nalesso et al. [35] have shown there is very little growth in the crystals after 5 s sonication. Therefore, the steeper gradients and shorter times to reach equilibrium in Fig. 7 highlights a higher crystal nucleation rate. Unlike the seed-similarity study in Section 3.2 designed to evaluate the initial nucleation induced, this study is designed to predominantly capture the secondary nucleation taking place. By using a much higher initial supersaturation ratio of 2.68 (compared to 1.03 used in the seed similarity study) and therefore a much higher driving force for nucleation, secondary nucleation is likely to dominate following the initial formation of primary nuclei, given the free-energy barrier for secondary nucleation to occur is much lower [4]. Secondary nucleation will continue until the system reaches saturation, which in this study is observed to occur within 90 s as can be seen in Fig. 7. Chirality-based crystal analysis, like that carried out in Section 3.2, was not possible in this study given the small crystal size and equilibrium reached following 90 s of sonication.

Fig. 7 shows the application of ultrasound significantly increases the crystallisation rate, with higher powers increasing it further. The increased rate with higher ultrasonic powers is in line with previously reported studies, where increased power is expected to result in more cavitation clouds leading to higher nucleation rates [8]. For both 98 kHz and 200 kHz, an almost identical trend was observed despite the 200 kHz having a slightly higher calorimetric power and cavitation activity as observed in Fig. 6. The rate improvements, and shorter time to reach equilibrium brought about by sonication compared to the silent, stirred case, are expected to be a result of significant nucleation rate improvements (ultrasound’s effect on improving the nucleation rate is widely reported [27], [28]).

### 3.4 Cavitation collapse thresholds for primary and secondary nucleation

Numerous studies have discussed ultrasound’s effect on primary nucleation by measuring bulk properties such as the induction time as an indicator of the extent to which ultrasound has an effect [20], [22], but to the authors’ best knowledge no study to date has categorically demonstrated that primary nucleation is taking place. While it has previously been theorised that the rate and other enhancements observed with sonocrystallisation could be a result of a secondary nucleation pathway [54], [55], the results from this study clearly demonstrates ultrasound’s ability to impact both primary nucleation and secondary nucleation.

Given the low supersaturation used in the seed similarity study (supersaturation ratio of 1.03), any primary nucleation occurring in the system is likely heterogeneous rather than homogeneous. Heterogeneous nucleation is able to occur at lower supersaturations as foreign solid surfaces are able to reduce the energy required for nucleation [4]. A model has been proposed in classical nucleation theory, where the decrease in free energy is dependent on a contact angle (or wetting angle) of the solid phase (an angle mathematically determined by resolving the interfacial tension forces between the foreign surface, the liquid and the crystalline deposit) [5]. This contact angle is directly used to calculate a correction factor which indicates the reduction in free energy required for heterogenous nucleation compared to homogenous nucleation to occur [5], [65].

It has been suggested that ultrasound is able to reduce the energy barrier or activation energy for crystallisation [20], [44]. According to Guo et al. and Wohlgemuth et al. this energy barrier is reduced as ultrasound is able to lower the contact angle and therefore the correction factor [44], [66]. Often proposed is the bubble as a nucleation centre theory [25], with Guo et al. suggesting that heterogeneous nucleation can take place at the cavitation bubble/solution interface [44]. That said, Guo et al. then suggests that it is unlikely nucleation at the bubble/solution interface would be a significant factor in reducing the contact angle [44] and have proposed that nucleation would more readily occur at the bubble/solution/foreign solid surface interface, whereby the bubble diameter and change in interfacial tension would be able to influence the contact angle [44]. It was also proposed that shockwaves, created following the implosion of a cavitation bubble, may induce forces on forming crystal nuclei, changing its shape and thereby reducing the contact angle allowing nucleation to more readily occur [44].

Although the above are proposed theories, they do suggest that a lower frequency would then favour primary nucleation as cavitation bubble size is inversely proportional to the applied frequency [67] and therefore lower frequencies would yield bubbles with a higher maximum radius and larger collapse intensity leading to shockwaves. Lower frequencies also typically result in increased transient cavitation (short-lived bubbles with high collapse intensities), whilst at higher frequencies stable cavitation bubbles (bubbles which undergo repetitive inertial collapse) with lower collapse intensities are typically generated [68], [69]. This may explain the greater reduction in seed similarity when applying the 98 kHz ultrasound compared to the 200 kHz ultrasound, suggesting more primary nucleation is induced at lower frequencies. It is possible there exists a collapse intensity threshold which must be overcome for primary nucleation to take place. A study by Lee and Yang, while not observing the same change in dominant primary nucleation mechanism from heterogeneous to homogeneous with increasing supersaturation, as seen in Guo et al.’s [44] and Lyczko et al.’s [20] studies, proposed an additional energy term which could be added to the classical nucleation equations to model the additional energy supplied by ultrasound [18]. The threshold required to induce primary nucleation is further supported by 98 kHz yielding a higher reduction in seed similarity despite having a lower cavitational activity (lower SL intensity in Fig. 5). This implies the importance of higher intensity cavitational collapse from individual bubbles at inducing primary nucleation rather than the overall number of cavitational events, which is much higher at 200 kHz (higher SL intensity in Fig. 5).

On the other hand, in the anti-solvent crystalisation system where secondary nucleation is expected to dominate, the same crystal nucleation rate was observed with both frequencies despite 98 kHz yielding a higher primary nucleation. This implies that a lower cavitational collapse intensity is needed to induce secondary nucleation compared to primary nucleation, and the number of cavitational events (more bubbles) experienced at higher frequencies then becomes important.

One would therefore expect the optimum ultrasonic frequency to be found in the low-mid kHz range, as is commonly reported in literature [8]. Depending on the crystallisation parameter studied, a slightly different optimum frequency may be found, given different ultrasonic frequencies may favour primary and secondary nucleation differently. Bulk properties such as induction time and MSZW evaluate the initial crystals which form focusing on primary nucleation (likely benefited by the lowest frequencies), while evaluating the time to reach a steady state crystal mass would likely focus on secondary nucleation (potentially benefited by slightly higher frequencies). This highlights the often discussed difficulty of comparing the effects of frequency on the crystallisation process [10], and the importance of deciding clear parameters to fix when comparing ultrasonic frequency, whether it be electrical input power (as was done in this study), or calorimetric power which, in this case, would have likely resulted in a higher rate at 98 kHz.

## 4 Conclusions

This study examined the extent to which ultrasound is able to induce primary and secondary nucleation. The results demonstrated conclusively that both 98 kHz and 200 kHz ultrasound was able to induce primary nucleation when stirring alone could not, with more primary nucleation observed at 98 kHz. While these results may have deviated from previous experiments which exclusively observed secondary nucleation, it is proposed that this was due to the previous methodology significantly favouring secondary nucleation and the increased sonication time leading to Viedma ripening, thereby clouding the previously reported results and conclusions. The short sonication time and “temporary” seeding used within this paper are more suited to study the initial nucleation induced by ultrasound, and by comparing to a non-sonicated but stirred system, a direct comparison can be made, allowing ultrasound’s ability to induce primary nucleation to be clearly demonstrated.

The anti-solvent crystallisation of sodium chlorate was also investigated finding similar rate enhancements with both 98 kHz and 200 kHz ultrasound. Given the higher initial supersaturation, secondary nucleation is expected to have dominated throughout the majority of the crystallisation process, and it would appear the more intense cavitational events which favour primary nucleation were not as important in inducing secondary nucleation, suggesting a lower cavitational collapse intensity threshold may exist for secondary nucleation to occur.

In the pharmaceutical industry it is often very important to control the chiral outcome during the crystallisation process. The reported results suggest that, despite seeding, short sonication times can result in significant variation in the chirality of the resulting crystals. While the application of ultrasound has significant benefits to increase the crystallisation rate as demonstrated through the anti-solvent study, the effects on both primary and secondary nucleation must be fully understood so the final chiral outcome can be readily predicted and controlled.

## CRediT authorship contribution statement

**Conor W. Copithorne-Crainey:** Conceptualization, Writing – original draft, Writing - review & editing, Visualization. **Fraser J. Armstrong:** Conceptualization, Writing – original draft, Writing - review & editing, Visualization. **Madeleine Bussemaker:** Writing - review & editing. **Judy Lee:** Conceptualization, Writing - review & editing, Visualization, Supervision, Project administration, Funding acquisition.

## Declaration of Competing Interest

The authors declare that they have no known competing financial interests or personal relationships that could have appeared to influence the work reported in this paper.
