# Supplementary material for: Use of enantiomeric properties of sodium chlorate to assess primary and secondary nucleation under sonication
Source: Ultrason Sonochem. 2021 Sep 22;79:105763. doi: 10.1016/j.ultsonch.2021.105763 (PMC8482043; doi:10.1016/j.ultsonch.2021.105763)
Supplement: Supplementary data 1 [file mmc1.docx]

**Supplementary Information for Influence of ultrasound on the primary and secondary nucleation rate of Sodium Chlorate**

Conor W. Copithorne-Crainey, Fraser J. Armstrong, Judy Lee

*Department of Chemical and Process Engineering, University of Surrey, Guildford, Surrey, GU2 7XH, United Kingdom*

The nucleation study was repeated with both left and right-handed crystals. Table S-1 summarises all the data collected for each nucleation experiment.

*Table S-1: Complete data, including seed crystal handedness, collected in the nucleation study.*

| **Seed Crystal handedness** | **Total Crystal mass (g)** | **Percentage Seed Similarity (%)** |
| --- | --- | --- |
| **Stirring + No US** | | |
| RH | 7.264 | 100.00 |
| RH | 8.879 | 100.00 |
| LH | 8.859 | 98.68 |
| RH | 7.257 | 99.09 |
| ***Average*** | ***8.332*** | ***99.26*** |
| ***Standard Deviation*** | ***0.929*** | ***0.66*** |
| **98kHz, 20W, No Stirring** | | |
| RH | 11.196 | 97.00 |
| LH | 9.519 | 67.90 |
| RH | 7.302 | 98.73 |
| LH | 8.704 | 94.39 |
| LH | 10.713 | 64.12 |
| ***Average*** | ***8.906*** | ***85.75*** |
| ***Standard Deviation*** | ***1.566*** | ***16.94*** |
| **200kHz, 20W, No stirring** | | |
| RH | 9.047 | 78.78 |
| LH | 10.368 | 92.90 |
| LH | 8.987 | 95.84 |
| RH | 7.574 | 98.09 |
| ***Average*** | ***8.994*** | **91.40** |
| ***Standard Deviation*** | ***1.141*** | ***8.68*** |
